# Third Space Endoscopic Therapies for Benign Motility Disorders: A Narrative Review

**DOI:** 10.1002/deo2.70397

**Published:** 2026-08-12

**Authors:** Sanjana Bhagwat, Amol Bapaye

**Affiliations:** ^1^ Shivanand Desai Center for Digestive Disorders Deenanath Mangeshkar Hospital and Research Center Pune India

**Keywords:** G‐POEM, peroral endoscopic myotomy, POEM, third‐space endoscopy, Z‐POEM

## Abstract

**Objectives:**

To review the evidence base, evolution, outcomes, adverse events (AEs), and management for third‐space endoscopy (TSE) procedures for management of benign motility disorders.

**Methods:**

This review examines TSE procedures for motility disorders, reviewing the current available techniques, success, outcomes, and long‐term outcomes from the literature.

**Results:**

Peroral endoscopic myotomy (POEM) for achalasia cardia is now established as first‐line therapy, endorsed by the American Society for Gastrointestinal Endoscopy, American College of Gastroenterology, Society of American Gastrointestinal and Endoscopic Surgeons, and international expert bodies, with long‐term clinical success exceeding 90% at 7 years. Since 2010, the technique has evolved considerably, with refinements in myotomy length, orientation, and depth, along with intraoperative use of an endoluminal functional lumen imaging probe to optimize outcomes and reduce post‐procedure gastroesophageal reflux (GER). The TSE platform has since expanded to spastic esophageal disorders, esophagogastric junction outflow obstruction, Zenker's and epiphrenic diverticula (Z‐POEM/D‐POEM), refractory gastroparesis (gastric per‐oral endoscopic pyloromyotomy), and Hirschsprung's disease. Techniques have also been modified along the way to achieve better results while minimizing AEs. Post‐POEM GER, occurring objectively in 40%–55% of patients, remains the principal long‐term challenge, and multiple modalities have been explored to address it.

**Conclusions:**

TSE has fundamentally transformed the management of benign gastrointestinal motility disorders, with POEM serving as the paradigm for the shift from surgical to endoscopic treatment. Ongoing technical refinement, GER mitigation strategies, and emerging innovations are poised to further advance the field.

**Trial Registration:**

N/A.

## 1 Introduction

Third‐space endoscopy (TSE), first described as submucosal endoscopy using a mucosal flap valve (SEMF), is based on the concept of accessing the deeper layers of the gastrointestinal (GI) tract through a submucosal tunnel while maintaining the mucosal integrity at the point of entry. TSE provides a protected working space for endoscopic myotomy and other interventions without risk of full‐thickness perforation. Sumiyama and colleagues first described this principle, and Pasricha et al. [1] demonstrated the feasibility of esophageal myotomy in an animal model [2]. Inoue's 2010 clinical series in human patients thereafter established peroral endoscopic myotomy (POEM) as a safe, reproducible, and highly effective procedure, and since then, its global dissemination has been rapid [3].

POEM is an established procedure for the management of achalasia cardia (AC) and is now the procedure of choice for a range of related esophageal motility disorders as well. The TSE technique has since been extrapolated for other spastic esophageal disorders (distal esophageal spasm [DES]; Jackhammer esophagus), esophagogastric junction outflow obstruction (EGJOO), Zenker's diverticulum (ZD), and epiphrenic diverticula (Z‐POEM/D‐POEM), refractory gastroparesis (gastric per‐oral endoscopic pyloromyotomy [G‐POEM]), and Hirschsprung's disease (per‐rectal endoscopic myotomy [PREM]) (Table 1). POEM was first reported in humans by Inoue et al. in 2010. Since then, POEM has been validated in multiple large prospective cohorts, randomized controlled trials, and meta‐analyses spanning over a decade. Studies have shown its efficacy to be comparable to laparoscopic Heller myotomy (LHM) for Types I and II achalasia and superior for Type III, with the advantages of tailoring myotomy length and shorter hospital stays. Multiple international societies—including the European, American, and Surgical guidelines, as well as the Indian expert consensus—have endorsed POEM as first‐line therapy where expertise is available [4, 5, 6, 7, 8].

**TABLE 1:** Overview of third space endoscopy procedures for benign motility disorders.

Procedure	Indication	Target structure	Clinical success	Key AEs
POEM (esophageal) [9, 10]	Achalasia Types I–III; Type III preferred	LES ± esophageal body	94% at 1 yr; 91.2% at 7 years	GER (40%–55%), mucosal injury, capnopneumothorax
POEM for Spastic Disorders [11, 12]	DES, Jackhammer esophagus, EGJOO	Esophageal body (15–20 cm) ± LES	DES 88%; JH 72%; EGJOO 93%	GER if LES included; mucosal injury
POEM for Sigmoid/End‐stage Achalasia [11, 13]	S1/S2 sigmoid achalasia, megaesophagus	LES (modified approach)	pooled 89.4%; 90.4%	AEs 3.6%–37.4%; greater technical difficulty
Z‐POEM [14, 15]	Zenker's diverticulum	Cricopharyngeal muscle/septum	Pooled – 90.6%; 93%; recurrence 6.7% at 37 months	AE 12.4%; lower recurrence versus FES
D‐POEM [15, 16]	Epiphrenic/mid‐esophageal diverticula	Diverticular septum ± esophageal muscle	Tech 95%–100%; Clinical 86%–97%	Similar to Z‐POEM
G‐POEM [17]	Refractory gastroparesis	Pyloric sphincter	Sham RCT: 71% versus 22%; meta‐analysis 61% at 1 year	Capnoperitoneum; abdominal pain
PREM [18, 19]	Hirschsprung's disease	Aganglionic rectal/sigmoid segment	High early success; comparable to surgery	Technically demanding; similar to POEM
POEM+F [20]	Achalasia + GER prevention intent	LES + endoscopic fundoplication	Achalasia 98.5%; objective GER 13.3% versus 58.3%	Major AEs 3.6%; no mortality

AEs, adverse events; DES, distal esophageal spasm; EGJOO, esophagogastric junction outflow obstruction; ES, Eckardt score; FES, flexible endoscopic septotomy; GER, gastroesophageal reflux; JH, Jackhammer esophagus; LES, lower esophageal sphincter.

Simultaneously, recognition of post‐POEM gastroesophageal reflux (GER) as the dominant long‐term limitation has spurred research into procedural modifications, such as shorter gastric myotomy to concomitant endoscopic fundoplication (POEM+F). This review evaluates the evidence, technical evolution, outcomes, and adverse‐event management for TSE procedures in benign GI motility disorders.

### 1.1 Technical Principles of TSE

**FIGURE 1:**
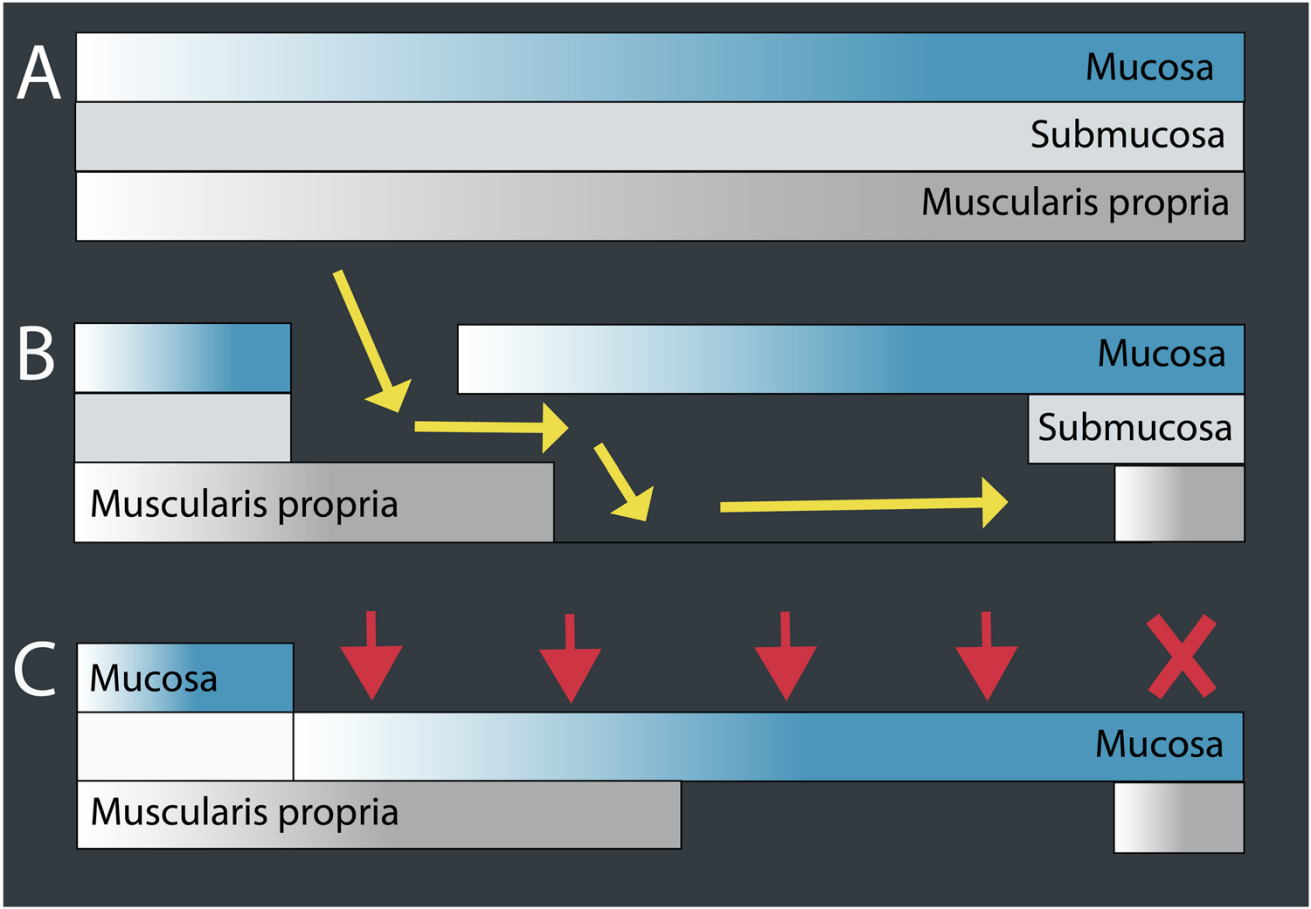
Principles of third‐space endoscopy (TSE). Line diagram describing the principle of TSE—(A) Normal three‐layer architecture of the gastrointestinal wall—mucosa, submucosa, and muscularis propria (B) Sequential steps of TSE: a mucosal entry point is created at a site separate from the target lesion, a submucosal tunnel is advanced through the third space to the target, endoscopic therapy (myotomy/resection) is performed at the target, and the tunnel is exited with closure limited to the mucosal entry site. (C) The mucosal flap‐valve concept: the intact mucosal–submucosal flap overlying the tunnel remains self‐sealing under intraluminal pressure (downward arrows) along its length, so only the entry incision—not the target/myotomy site (X)—requires endoscopic closure.

All TSE procedures share four fundamental operative steps: 1] mucosal incision—a submucosal bleb is raised with a viscous lifting agent and a 1.5–2 cm longitudinal incision provides tunnel entry; 2] submucosal tunneling—the endoscope advances within the submucosal plane past the target structure using injection‐dissection technique; 3] deep‐layer intervention—myotomy, septotomy, or other specific intervention on the muscularis propria or septum; 4] mucosal closure—endoscopic clips, suturing, or tacking devices close the entry site securely (Figure 1).

**FIGURE 2:**
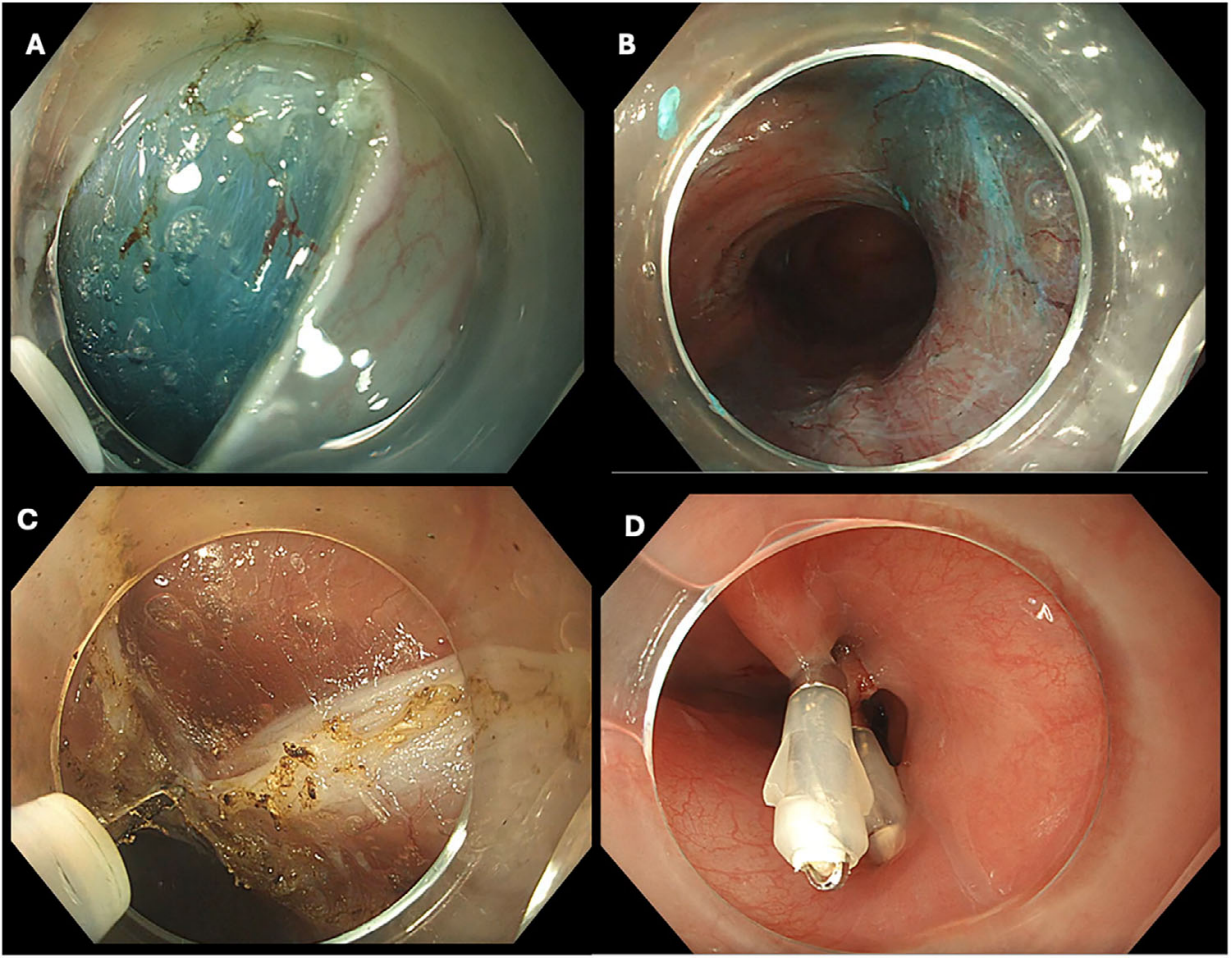
Per‐oral endoscopic myotomy. (A) Submucosal injection of dilute methylene blue, mucosal incision proximal to the gastroesophageal junction followed by creation of submucosal tunnel. (B) Completed submucosal tunnel. (C) Myotomy being performed. (D) Closure of the original esophageal mucosotomy (tunnel entry site) with through‐the‐scope clips.

Carbon dioxide insufflation is mandatory throughout all TSE procedures. General anesthesia with endotracheal intubation is universal. Familiarity with electrosurgical generator settings and different coagulation and cut modes is essential. The choice of knife (triangular‐tip, Hybrid knife, IT knife) and injection solution (normal saline, Hetastarch, or hydroxypropyl methylcellulose with dye) varies by operator preference and available resources.

## 2 POEM for Achalasia Cardia: An Established Standard

### 2.1 Outcomes and Long‐Term Durability

POEM has demonstrated consistently high clinical success rates across large multicenter and single‐center series [9] (Figure 2 and Table 2). A 10‐year sequential cohort from Modayil et al. (610 patients) showed sustained success rates at 91.2% at 7 years—remarkable durability for any endoscopic therapy [10]. For Type III achalasia, POEM is superior to LHM: a multicenter study demonstrated 98% versus 80.8% clinical response (*p* = 0.01), attributable to POEM's capacity for longer tailored myotomy [11]. The Werner et al. randomized controlled trial confirmed POEM is non‐inferior to LHM+Dor fundoplication at 2 years for all achalasia types, though with significantly higher rates of objective GER [12].

**TABLE 2:** Key long‐term peroral endoscopic myotomy (POEM) outcome studies.

Study	*N*	Follow‐up	Pre‐ES	Post‐ES	Success%
Modayil et al. (10‐year sequential cohort) [22]	610	Up to 7 years	7.6	0.5–1.2	91.2 at 7 years
Li et al. (5‐year follow‐up) [26]	564	49 months	8	2	87.1 at 5 years
Shiwaku et al. (multicenter, Japan) [10]	1346	12 months	6.1	1.1	94.7
Hasan et al. GIE 2022 (technical review) [25]	Review	—	—	—	90%–98% consistently

ES, Eckardt score. Clinical success defined as ES ≤3 unless otherwise stated.

Hasan et al. provide an extensive evidence‐based review of technical adaptations since POEM's inception. Since the technique was first standardized, adaptations in myotomy location, length, and thickness have been pursued to optimize efficacy and minimize GER without sacrificing clinical success. These are discussed in the technical evolution section below.

### 2.2 Technical Evolution of POEM: Anti‐Reflux Refinements

Post‐POEM GER, occurring objectively in 14 %–57 % of patients [9], has been the primary driver of technical refinement since POEM's introduction. Multiple modifications have been investigated to preserve efficacy while reducing reflux burden.

#### 2.2.1 Myotomy Length

A short esophageal myotomy for Types I and II achalasia achieves clinical outcomes similar to those of longer myotomies up to 10 cm, with reduced procedure time, as confirmed by multiple randomized controlled trials and meta‐analyses [13, 14]. For gastric extension, limiting the myotomy to 2–2.5 cm reduces the risk of reflux while normalizing EGJ distensibility [15].

#### 2.2.2 Myotomy Depth: Selective Circular Muscle Myotomy

Selective circular muscle myotomy—preserving the longitudinal muscle layer—has been shown in a few studies to be associated with reduced post‐procedure reflux symptoms and erosive esophagitis, without affecting treatment success or adverse events (AEs). A recent meta‐analysis showed that although GER rates are higher with full‐thickness myotomy, the association with erosive esophagitis is not significant in the presence of consistent proton‐pump inhibitor (PPI) use [16].

#### 2.2.3 Myotomy Orientation: Anterior Versus Posterior

Both anterior (1–2 o'clock) and posterior (5–6 o'clock) POEM approaches achieve equivalent clinical success for achalasia. There is a trend toward higher post‐POEM esophageal acid exposure with the posterior approach; a randomized controlled trial comparing the two approaches found no significant difference in the clinical efficacy or the occurrence of post‐POEM GER [17].

#### 2.2.4 Sling Fiber Preservation

The EGJ anti‐reflux barrier comprises the internal lower esophageal sphincter (LES)—circular clasp fibers and oblique sling fibers—and the external LES (crural diaphragm). A recent online abstract by Shiwaku et al. [18] showed that intentional preservation of the oblique sling fibers during posterior POEM significantly reduced severe (LA grade ≥ C) erosive esophagitis (18.5% vs. 44.1% in conventional POEM). The technique identifies two penetrating vessels from the left gastric artery at the cardia as anatomical landmarks: myotomy is directed to the right of the second penetrating vessel (toward the lesser curvature), sparing the sling fibers. Double‐scope transillumination can be used to confirm the tunnel's correct orientation. Tanaka et al. reported similar findings, with grade B or higher esophagitis 31.3% versus 58.1% (*p* = 0.017) [19]. However, in a randomized controlled trial by Nabi et al., oblique fiber‐sparing myotomy showed no significant reduction in reflux esophagitis, acid exposure time (AET), or DeMeester score versus conventional myotomy—making routine adoption operator‐dependent pending further evidence [20].

#### 2.2.5 Endoluminal Functional Lumen Imaging Probe‐guided Myotomy

The endoluminal functional lumen imaging probe (EndoFLIP) measures pyloric and EGJ cross‐sectional area (CSA), intraluminal pressure, and distensibility index (DI) in real time. A pre‐procedure DI <6 mm^2^/mmHg is predictive of lower post‐POEM GER risk; post‐myotomy DI and CSA confirm adequate myotomy completion and help guide incremental myotomy length. Knight et al. [21] reported that EndoFLIP‐tailored POEM in two UK centers enabled shorter myotomies without compromising clinical success, while intraoperative DI and final CSA were significantly associated with postoperative esophagitis on follow‐up EGD. A systematic review and meta‐analysis confirmed that EndoFLIP use during POEM is associated with physiologically optimized outcomes for both POEM and LHM, with pre‐to‐post change in DI being more informative than a single intraoperative measurement [22].

## 3 Post‐POEM GER: The Major Long‐Term Challenge

GER is the most significant long‐term concern following POEM. Post‐POEM GER has been reported at 9%–43% (symptomatic GER disease [symptomatic GERD]), 13%–68% (reflux esophagitis), and 38%–57% (positive pH studies) across published series [23]. The discrepancy between symptomatic and objective reflux rates is a defining feature of post‐POEM GER: pathologic acid exposure is far more common than symptomatic GERD, and subjective quality‐of‐life measures do not reliably predict objective esophageal acid burden. Additionally, fermentation of undigested food due to stasis in the dilated esophagus can lead to false‐positive pH study results; accurate diagnosis requires manual pH trace review and application of the updated Lyon Consensus 2.0 criteria, which designate LA grade B or higher esophagitis and AET >6% as conclusive evidence of GERD.

Risk factors for post‐POEM GER include age >65 years, BMI >35 kg/m^2^, hiatal hernia, full‐thickness myotomy (odds ratio 3.99), gastric myotomy >2 cm, and esophageal myotomy >10 cm. Unlike LHM—where an anti‐reflux procedure (partial fundoplication) is routinely added—standard POEM does not include fundoplication. Erosive esophagitis with progression to Barrett's metaplasia, though rare, has been documented after POEM, emphasizing the need for long‐term endoscopic surveillance [24].

### 3.1 Management of Post‐POEM GER

The first‐line therapy for post‐POEM GER is PPI therapy, which achieves symptomatic resolution in 66%–100% of patients. PPI therapy can also lead to resolution of erosive GERD; however, optimal duration remains undefined. CYP2C19 pharmacogenetic variability may account for approximately 6.8% of PPI‐refractory cases. Rabeprazole, being less affected by CYP2C19 metabolism, may be preferred in these scenarios. Management options range from PPI dose optimization to endoscopic fundoplication (POEM+F, TIF) and laparoscopic fundoplication for PPI‐refractory cases. Other investigational approaches, including Stretta, magnetic sphincter augmentation, electrical stimulation, and anti‐reflux mucosectomy, require further study in the post‐POEM context.

### 3.2 POEM With Concomitant Endoscopic Fundoplication

**FIGURE 3:**
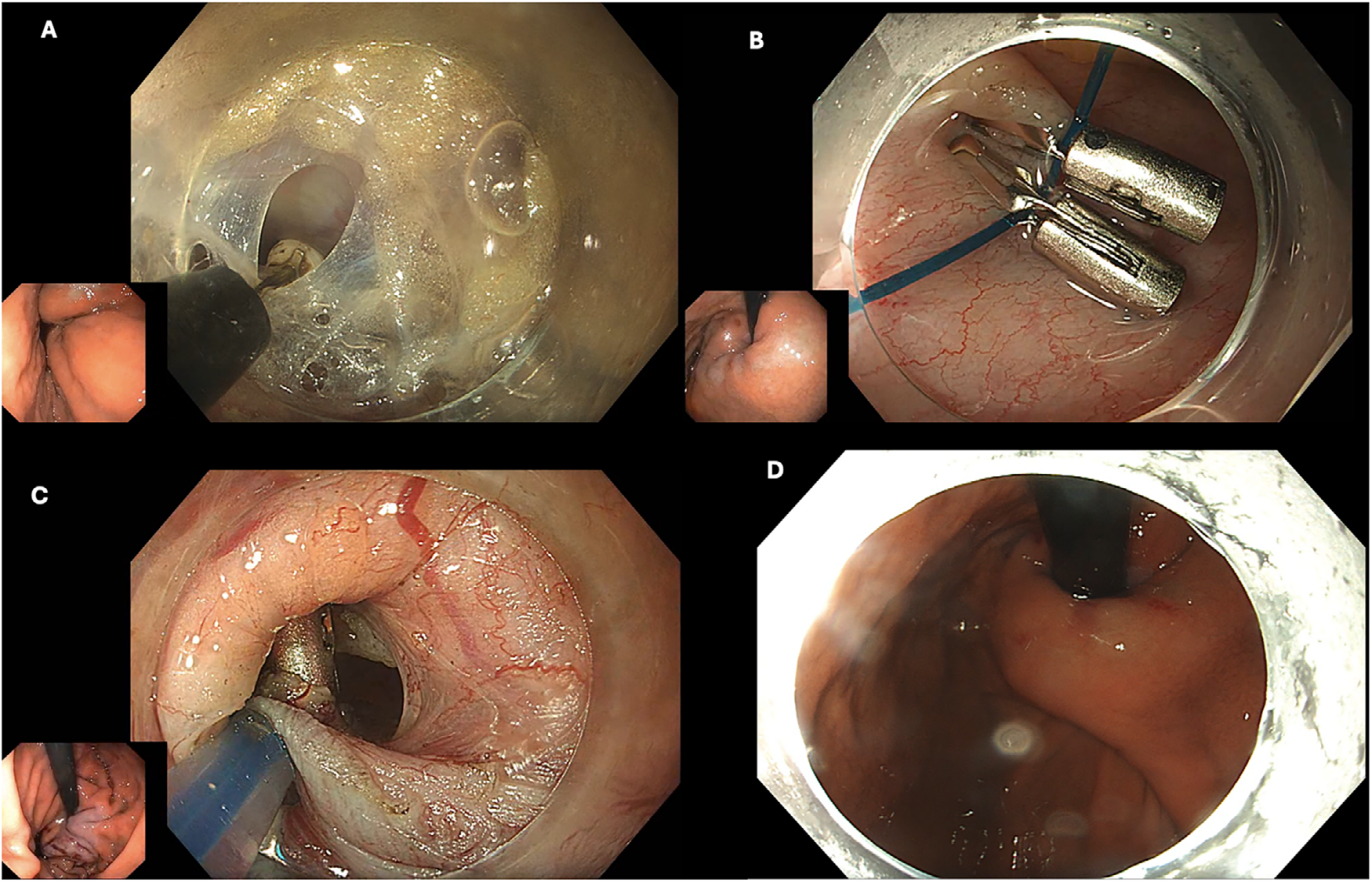
Fundoplication concomitant with POEM for preventing reflux (POEM+F). (A) Peritoneal entry using IT2 knife after completion of myotomy. Retroflexed gastric view of the GEJ/cardia (inset). (B) Placement of through‐the‐scope clips with endoloop at a prior marked position on the gastric fundus on the peritoneal aspect (inset: second scope showing the GE junction in retroflexion). (C) Tightening of the endoloop after securing the other end within the tunnel creating a partial wrap (inset—creation of wrap seen on retroflexed view with second scope). (D) Well‐formed fundoplication wrap seen on retroflexion. (H), completing the POEM+F procedure.

Inoue first described endoscopic fundoplication concomitant with POEM—performed as a pure NOTES procedure—to simulate Dor's partial anterior fundoplication [25]. After standard anterior POEM with full‐thickness gastric myotomy, the endoscope is passed into the peritoneal cavity through the myotomy tunnel; an endoloop is anchored to the gastric fundus and tunnel edge via endoclips, which creates an anterior fundic wrap. (Figure 3) Bapaye et al. reported 1‐year outcomes: 92% technical success, wrap integrity 82.6%, and objective GER (abnormal AET by Lyon criteria) in only 11.1% [26]. A subsequent 3‐year matched cohort study showed significantly lower objective GER in the POEM+F group (13.3% vs. 58.3%, *p* = 0.037) with wrap integrity in 76.5% at 3 years and comparable clinical success in both groups [27] (Table 3). A recent online abstract reported favorable outcomes at 5‐year follow‐up after POEM+F, with sustained clinical outcomes and no significant deterioration in GER‐related outcomes [28].

**TABLE 3:** Comparative outcomes of peroral endoscopic myotomy (POEM) versus POEM+F at 3 years.

Parameter	POEM+F	POEM alone	*p*‐Value
Clinical success (ES <3)	98.5%	98.5%	NS
Objective GER (Lyon Consensus 2.0)	13.3%	58.3%	0.037
Symptomatic GER (GERD‐Q)	11.7%	17.6%	NS
Wrap integrity at 3 years	76.5%	N/A	—
Regular PPI use	5.9%	29.4%	0.04
Major adverse events	None	None	—

Bapaye et al. *Gastrointest Endosc*. 2025. Matched cohort, *n* = 34 per group. ES, Eckardt score; GER, gastroesophageal reflux; NS, not significant.

These single‐center findings are now supported by a systematic review and meta‐analysis by Gopakumar et al. [29], which pooled data from seven studies (127 patients). Pooled technical success of POEM was 96.9%; of fundoplication, 92.3%; achalasia clinical success 96.4%; wrap integrity 84.0%; composite GER control 86.2% (95% confidence interval 73.8–93.2); abnormal pH monitoring in 21.8%; LA grade B or higher esophagitis in 14.4%; and major AEs in 3.6%, with no procedure‐related mortality. The first US experience was reported by Shrigiriwar et al. [30]. The technique remains operator‐dependent; hiatal hernia, sigmoid achalasia, and extremely fibrosed submucosa are relative contraindications. A well‐powered randomized controlled trial comparing POEM+F with POEM is awaited.

## 4 POEM For Non‐Achalasia Esophageal Motility Disorders

POEM can deliver a precisely tailored myotomy of any length under direct visualization, making it an attractive option for non‐achalasia esophageal motility disorders (NAEMDs). POEM for NAEMDs differs substantially from achalasia POEM: myotomy length must be customized to the manometric pattern, LES involvement is selective, technical difficulty is higher due to thickened spastic muscle, and patient selection is considerably more complex. A systematic multidisciplinary pre‐procedure evaluation—incorporating HRM (Chicago Classification v4.0), timed barium esophagram, EndoFLIP, and upper GI endoscopy—is mandatory.

### 4.1 DES and Jackhammer Esophagus

The technical success and clinical efficacy of POEM in spastic disorders have been reported in multiple studies and meta‐analyses [31, 32, 33]. DES (premature contractions, DL <4.5 s in ≥20% swallows, normal IRP) and hypercontractile (Jackhammer) esophagus (DCI > 8000 mmHg·s·cm in ≥20% swallows) frequently require extended esophageal body myotomies of 15–20 cm tailored to the proximal limit of abnormal contractions on HRM. Administration of intravenous nitroglycerin (100–200 mcg) during POEM for DES can relax intraprocedural spasms and facilitate tunneling. LES myotomy inclusion is recommended for Jackhammer esophagus to prevent iatrogenic functional obstruction from the long body myotomy. In a meta‐analysis of nine studies and a cohort of 210 patients, pooled clinical success rates are 88% for DES and 72% for Jackhammer esophagus [34]. Large retrospective series report 98.1% clinical success at 2–3 months, declining to 92.6% at 1 year [32].

### 4.2 Esophagogastric Junction Outflow Obstruction

EGJOO accounts for 3%–21% of clinical HRM studies, though a significant proportion of these studies represent functional, self‐resolving, or secondary conditions that require rigorous exclusion. Ichkhanian et al. [35] reported 93% clinical success at 6 months; however, erosive esophagitis developed in 50% of patients, reflecting a high risk of GER when the intact LES is completely disrupted. Longer follow‐up shows progressive symptom recurrence, consistent with the fluctuating natural history of EGJOO.

### 4.3 Redo‐POEM and Sigmoid/End‐Stage Achalasia

For persistent or recurrent achalasia after prior POEM or LHM, repeat POEM (using contralateral tunnel orientation) is preferred over pneumatic dilation: a multicenter RCT of 90 patients demonstrated POEM success of 62.2% versus 26.7% for PD. For sigmoid‐type achalasia, a dedicated meta‐analysis (eight studies, 248 patients) showed pooled clinical success of 90.4%, technical success 98.3%, and objective GER of 41.5% [36, 37]. A recent abstract online also showed efficacy of POEM+F in patients with sigmoid esophagus [38].

## 5 Diverticular Myotomy: Z‐POEM And D‐POEM

### 5.1 Z‐POEM for ZD

**FIGURE 4:**
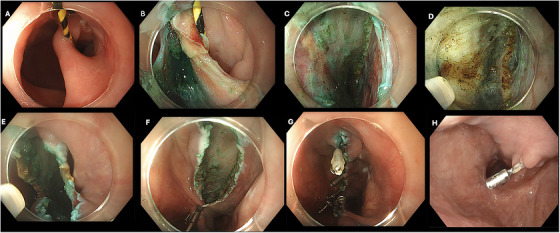
Zenker's per‐oral endoscopic myotomy (Z‐POEM). (A) Endoscopic view of the esophageal lumen (guidewire within lumen) and the Zenker's diverticulum. (B) Submucosal injection followed by transverse incision over the bridge. (C, D) Mucosotomy and creation of the submucosal tunnel along the septum on both sides, exposing the septal/cricopharyngeal muscle fibers. (E, F) Selective myotomy of the septal muscle (cricopharyngeus and adjoining circular fibers) performed within the tunnel up to the base of the diverticulum. (G) Closure of the mucosal entry site with through‐the‐scope clips. (H) Endoscopic appearance on follow‐up showing obliteration of the septum and opened up diverticulum with no stasis.

ZD is a pharyngoesophageal diverticulum, for which the treatment approach has shifted from traditional open surgery to minimally invasive rigid or flexible endoscopic techniques. Z‐POEM (submucosal tunneling endoscopic septum division), first described by Li et al., performs cricopharyngeal myotomy within a protected submucosal tunnel—enabling more complete septotomy to the diverticular base than flexible endoscopic septotomy (FES), which carries 11%–15% recurrence [39]. The technical challenges for performing septotomy include limited working space and anatomical constraints. The standard Z‐POEM technique involves dual tunneling followed by septotomy, and the incision is then closed with clips. This technique is complex and challenging, and since its first description, various technical modifications have been reported, including single‐tunnel Z‐POEM, tunnel‐free Z‐POEM, precut myotomy, open Z‐POEM, and non‐injection variants (Figure 4). Thus, no standardized protocol currently exists, and critical evidence on these evolving techniques is essential. The size of the ZD may also influence the technique selection [40].

Long‐term data from a multicenter international cohort (14) in 89 patients demonstrated 94% clinical success and recurrence in only 6.7%—substantially lower than the historical FES recurrence rate of 11%–15%. A systematic review and meta‐analysis (11 studies, 357 patients) confirmed a pooled clinical success of 93% and an AE rate of 12.4% [41]. Technical modifications described by different studies are mentioned in Table 4.

**TABLE 4:** Endoscopic management of Zenker's diverticulum—technique comparison.

Technique	Technical success	Clinical success	Recurrence	Adverse events	Key evidence
FESD/FED (flexible endoscopic septum division)	∼99%	91% pooled (range 90%–100%); ∼94% for standard FESD	∼11%	11.3% pooled (bleeding ∼6.6%, perforation ∼5.3%)	Ishaq et al. (20 studies, 813 pts) [54]; Deepanshu et al. (997 pts) [55]
Z‐POEM/STESD (Zenker peroral endoscopic myotomy)	96.3%–97.3% pooled rates	92%–93% pooled rates	11.2% pooled (2‐yr ∼6.7%)	12.4% pooled (6.7% in Yang cohort)	Zhang H. meta‐analysis (11 studies, 357 pts) [53]; Yang multicenter international study (75 pts) [56] Facciorusso meta‐analysis (300 pts) [15]
POES (peroral endoscopic septotomy)	95%–97%	89%–91%	8%–12%	16%–17%	Spadaccini meta‐analysis (POES *n* = 63); [57] Mittal (*n* = 24);
POED (peroral endoscopic diverticulotomy—hybrid, injection‐guided)	100%	100%	None reported	No major AEs (small series)	Pugliese case series (5 pts) [58]—early/limited data
RES (rigid endoscopic septotomy)	79%–88%	70%–89%	9%–26%	5%–30%	Cadena Aguirre meta‐analysis (*n* = 453) [59]

### 5.2 D‐POEM for Epiphrenic and Mid‐Esophageal Diverticula

D‐POEM follows the principle of Z‐POEM and is performed for epiphrenic or mid‐esophageal diverticula. These are commonly seen to occur in association with achalasia cardia or other esophageal motility disorders. It has the added advantage of treating the underlying motility disorder along with the diverticulum, as failure to address the concurrent disorder is a major contributor to recurrence. combines septotomy of the diverticular neck with myotomy of any concurrent esophageal motility disorder (frequently DES or achalasia) in the same session, with technical and clinical success of 95%–100% and 86%–97%, respectively [42, 43, 44].

### 5.3 G‐POEM For Gastroparesis

**FIGURE 5:**
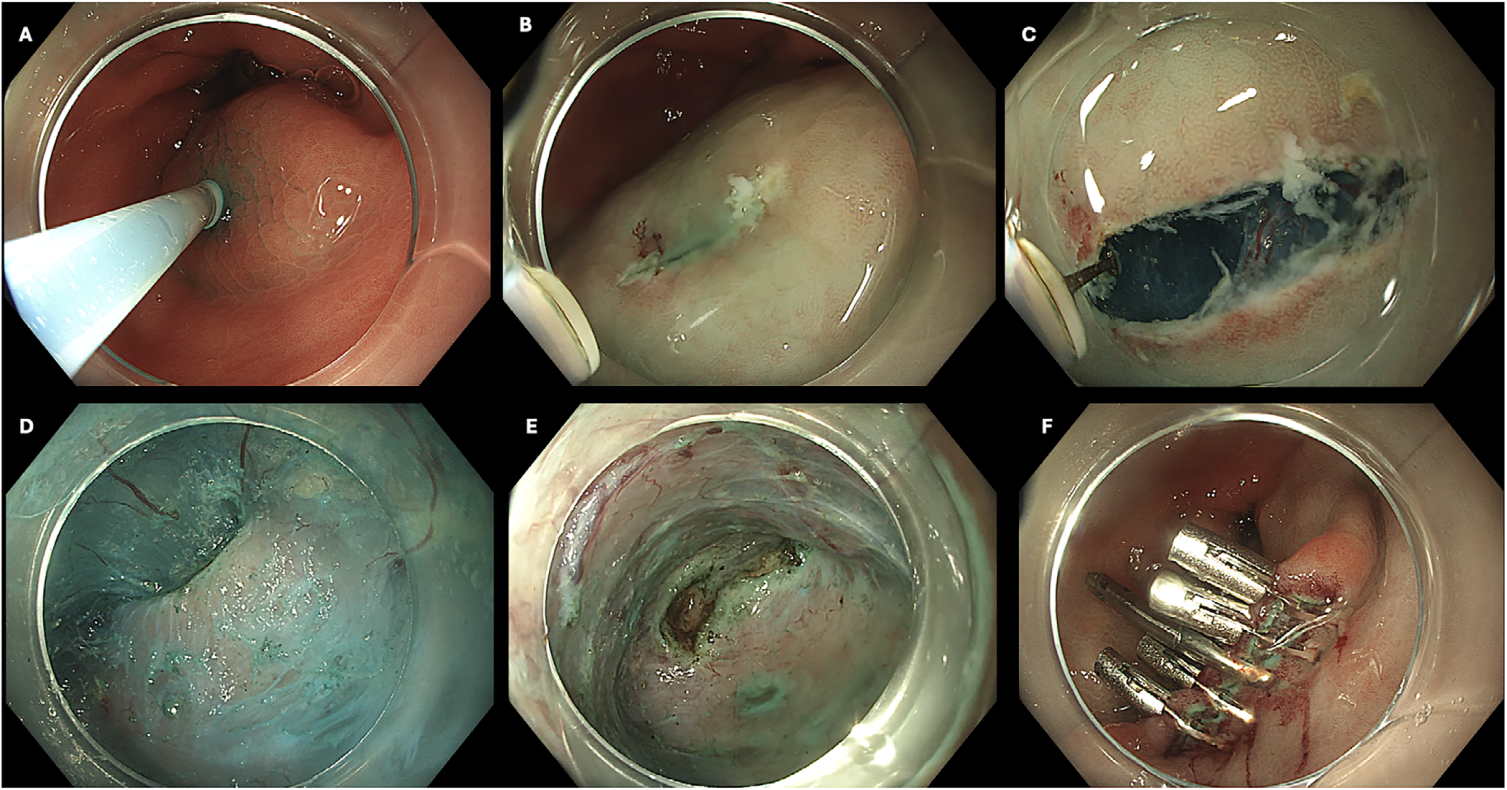
Gastric per‐oral endoscopic pyloromyotomy (G‐POEM). (A) Antral view demonstrating the pylorus and submucosal injection of dilute methylene blue being performed proximal to the pylorus (4‐5 cm proximal). (B) Mucosal incision on the antral mucosa proximal to the pyloric ring to establish the mucosal entry point. (C) Submucosal dissection and creation of the tunnel. (D) Submucosal tunnel advanced across the pyloric ring into the duodenal bulb, exposing the pyloric muscle. (E) Short pyloromyotomy (about 2 cm) in distal to proximal direction. (F) Closure of the mucosal entry site with through‐the‐scope clips.

G‐POEM, first performed in humans by Khashab et al. [45], applies the SEMF principle to the pyloric sphincter (Figure 5). It targets pyloric dysfunction—impaired relaxation, reduced distensibility, and increased tone—contributing to delayed gastric emptying in a pathophysiologically important subset of gastroparesis patients. The standard technique employs mucosal entry 4–5 cm proximal to the pylorus; submucosal tunneling toward the pyloric ring; full‐thickness pyloromyotomy of 2–3.5 cm in distal‐to‐proximal direction; and mucosal closure (OverStitch preferred over clips for thicker gastric mucosa). The standard approach is from the greater curvature; however, some operators prefer the lesser curvature, rarely the anterior or the posterior wall [46]. Thus, in medically refractory disease, G‐POEM is recommended after careful patient selection. Thus, in medically refractory disease, G‐POEM can be performed after careful patient selection.

#### 5.3.1 Technical Variants

Antro‐pyloromyotomy extends the myotomy 2–3 cm proximal to the pylorus through antral musculature (total 4–6 cm) to address the component of antral hypomotility, with reported 80% clinical success at 6 months [47]. Double pyloromyotomy— a second parallel myotomy adjacent to the first— demonstrated superior 6‐month clinical response (86% vs. 67% single myotomy) [48].

### 5.4 EndoFLIP and Patient Selection

Patient selection is the central challenge in G‐POEM; the 1‐year success rate is only 61% [49]; 60% show a lower risk of recurrence [44]. EndoFLIP provides dynamic physiological characterization of pyloric function, with a pre‐procedure DI threshold of 9.2 mm^2^/mmHg identified as predictive of clinical success (100% specificity, 72% sensitivity) [50].

### 5.5 Clinical Outcomes and Evidence‐Based

Technical success is consistently >95%. The pivotal sham‐controlled RCT (Martinek et al., Gut 2022; 46 centers, 279 patients) demonstrated a clinical response rate of 71% in G‐ POEM versus sham 22% (*p* < 0.001)—the first level 1 evidence confirming a specific therapeutic effect beyond placebo [51]. The international prospective multicenter trial reported clinical success of 52% at 6 months [52]. A meta‐analysis (10 studies, 482 patients) reported a pooled clinical success of 61% at 1 year, with AEs in 8% [53]. Long‐term data show that at 1 year, 85.2% had sustained response. The study reported a 12.9% annual loss in response rate after the initial clinical response [54]. The fundamental limitation is that gastroparesis is a syndrome—not a single disease—and pyloric dysfunction is only one of several pathophysiological mechanisms. Robust pre‐operative phenotyping integrating EndoFLIP, gastric emptying scintigraphy, antro‐duodenal manometry, and botulinum toxin response testing remains the principal unmet need.

## 6 PREM For Hirschsprung's Disease

**FIGURE 6:**
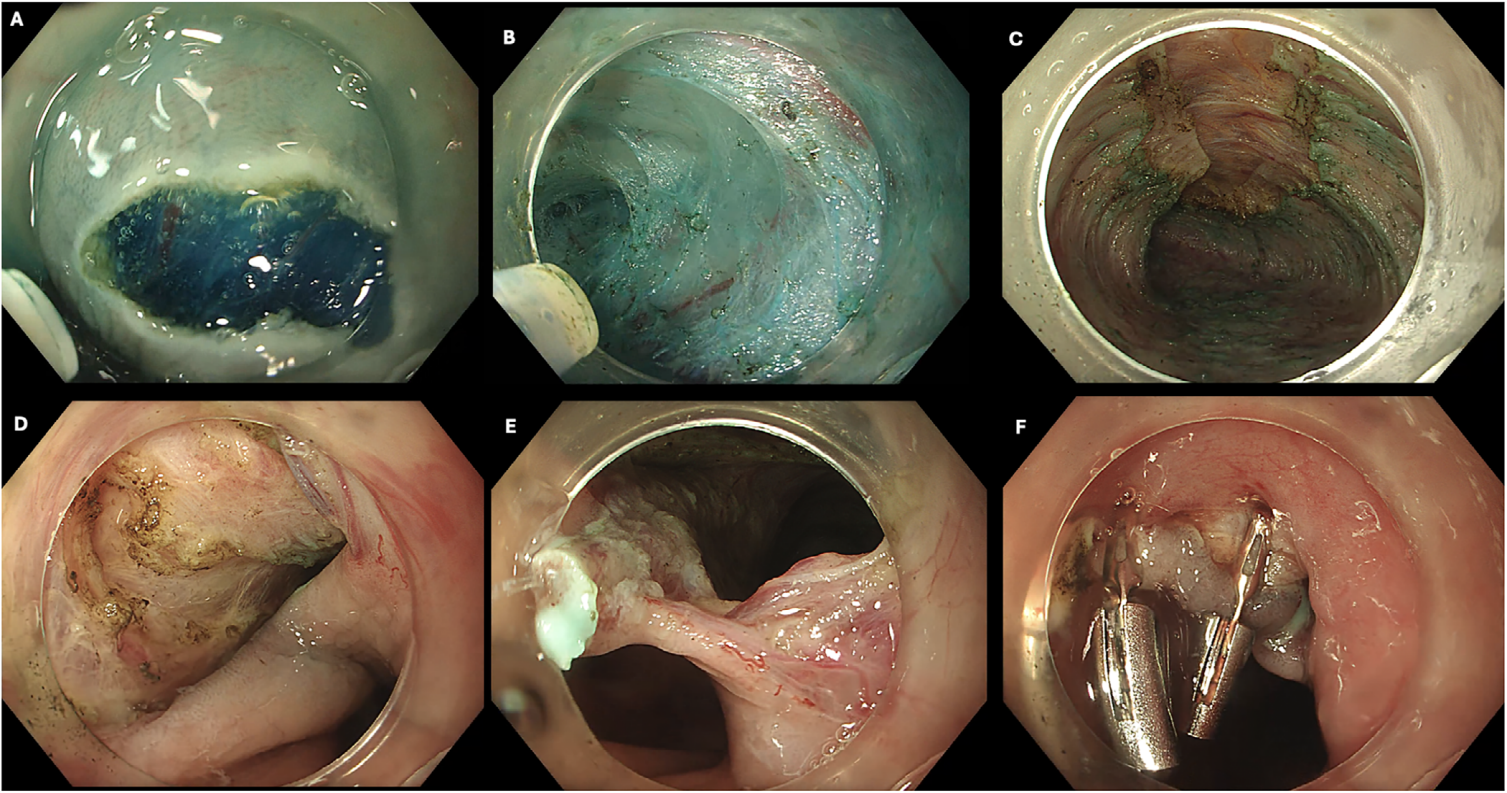
Per‐rectal endoscopic myotomy (PREM) for Hirschsprung's disease. (A) Submucosal injection and incision just inside the anorectal junction. (B) Submucosal tunnel created in ano‐oral direction for predetermined length based on pre‐procedure mapping of aganglionic segment by immunohistochemistry. (C) Full‐thickness myotomy in the oro‐anal direction. (D) Myotomy including the internal anal sphincter. (E) Completed myotomy. (F) Closure of the rectal mucosotomy with through‐the‐scope clips.

PREM applies the SEMF principle to the rectum and sigmoid, creating a submucosal tunnel through the rectal mucosa and performing myotomy of the aganglionic segment (Figure 6). It is primarily indicated for short‐segment residual Hirschsprung's disease or acquired megacolon, post‐pull‐through, or for recurrent constipation after surgical correction. Accurate preoperative mapping of the aganglionic segment length is mandatory as it will dictate the myotomy and tunnel length. This can be done with suction EMR deep biopsies that capture the submucosal layer, allowing histologic localization of the transition zone. Technical success rates exceed 90%, with promising early clinical outcomes comparable to those of surgical redo pull‐through, avoiding the need for laparotomy. The procedure is highly technically demanding in the rectal working space and is best reserved for specialized TSE centers. The most informative dataset is by Dr. Bapaye et al., which demonstrates effective medium‐ and long‐term outcomes [55, 56, 57]. A recent international multicenter study published in abstract form showed efficacy with PREM for 52 patients with acquired megacolon or Hirschsprung's disease with significant improvement in unit laxative use pre‐PREM = 4.3 U [±2.5] to post = 1.6 U [±0.9], [*p* < 0.0001]) and stool frequency (pre‐PREM once every 7.8 days [±4.3] to post—once every 1.3 days [±0.6] [*p* < 0.0001]) [58].

Table 6 summarizes the outcomes of TSE for indications.

## 7 AEs: Prevention, Recognition, And Management

AEs during TSE follow a characteristic and largely predictable pattern determined by the shared SEMF technique. Three major categories are recognized: (1) insufflation‐related AEs; (2) mucosal injuries; (3) bleeding. Additional relevant AEs include infectious complications, aspiration pneumonia, chest or abdominal pain, atelectasis, cardiac arrhythmias, pleural effusion, and pulmonary embolism. Yewale et al. provide a comprehensive framework for prevention, early detection, and management of TSE‐specific AEs. (Table 5) [59].

**TABLE 5:** Spectrum of adverse events in third space endoscopy.

Adverse event	Category	Incidence	Management
Mucosal injury (mucosotomy)	Intra‐procedural	3.9%–10%	Endoclip closure during POEM; larger defects may need suturing
Capnothorax/capnomediastinum	Intra‐procedural	Clinically significant ∼1%–5%	14G angiocath decompression; conservative; CO_2_ resolves rapidly
Tension capnoperitoneum	Intra‐procedural	∼1%–3%	Immediate 14G angiocath, right upper quadrant; PIP monitoring
Intraprocedural bleeding	Intra‐procedural	Major <1%; minor frequent	Coagulation grasper (soft coag); irrigation; cap tamponade
Pleural effusion	Post‐procedural	∼1%	Conservative: chest drain if symptomatic/large
Aspiration pneumonia	Post‐procedural	∼1%	Antibiotics; respiratory support; nasogastric decompression pre‐POEM
Delayed bleeding/tunnel hematoma	Post‐procedural	<1%	Re‐exploration; coagulation; lavage; clips if needed
Post‐POEM GER/erosive esophagitis	Long‐term	Objective 40%–55%; symptomatic 15%–40%	PPI; Lyon 2.0 criteria for diagnosis; POEM+F or TIF for refractory
Barrett's metaplasia/adenocarcinoma	Long‐term	Rare; case reports	Long‐term surveillance endoscopy; mandatory post‐POEM follow‐up

AET, acid exposure time; LA, Los Angeles classification; PPI, proton pump inhibitor.

**TABLE 6:** Outcomes of expanded third‐space endoscopy (TSE) applications for non‐achalasia motility disorders.

Procedure/Indication	*N*/Studies	Technical success	Clinical success	Key notes
G‐POEM (gastroparesis)	Sham‐RCT (Martinek, Gut 2022); multicenter (Vosoughi, Gut 2022; 46 centers, 279 pts)	>95%	71% versus sham 22% (6 mo); meta‐analysis 61% at 1 year	Sham RCT = level 1 evidence; EndoFLIP DI <9.2 mm^2^/mmHg predictive;
G‐POEM (double pyloromyotomy)	Abdelfatah et al. GIE 2020; 90 pts	>95%	86% versus 67% single (6 mo)	No added complexity; double pyloromyotomy promising
Z‐POEM (Zenker's)	Meta‐analysis: 11 studies, 357 pts; multicenter 89 pts	96.3%	93%; recurrence 11.2% pooled, 6.7% at 37 months	Open Z‐POEM (OZ‐POEM)—entry site left open, reduced recurrence; TPV as landmark
POEM DES / Jackhammer	Meta‐analysis: nine studies, 210 pts	N/A	DES 88%; JH 72%	Long myotomy 15–20 cm; LES preservation reduces GER; opioid exclusion mandatory
POEM EGJOO	Ichkhanian et al.; 15 pts prospective	100%	93% at 6 months	Esophagitis 50% if LES included; secondary causes must be excluded; FLIP confirms physiology
Repeat POEM (failed myotomy)	Multicenter RCT 90 pts	N/A	POEM 62.2% versus PD 26.7%	Contralateral orientation avoids fibrosis; POEM superior to PD after failed Heller
PREM (Hirschsprung's)	Small series	>90%	Comparable to surgical pull‐through	Pediatric; short‐segment HD; technically demanding; expert centers only

AEs, adverse events; DES, distal esophageal spasm; EGJOO, esophagogastric junction outflow obstruction; JH, Jackhammer esophagus; PD, pneumatic dilation; RCT, randomized controlled trial.

### 7.1 Insufflation‐Related AEs

CO_2_ insufflation within the confined submucosal tunnel can generate gas‐related AEs. Gas‐related AEs include capnomediastinum, capnothorax, and capnoperitoneum. Mild mediastinal or subcutaneous emphysema is universal and clinically inconsequential; tension capnoperitoneum is the most feared acute AE. Vigilant monitoring of peak inspiratory pressure (PIP) by the anesthesiologist is the first alarm; external abdominal palpation confirms a tense abdomen; and a 14‐G angiocath placed percutaneously in the right upper quadrant provides immediate decompression, rapidly normalizing PIP and hemodynamics. CO_2_ use mandatorily reduces the risk of clinically significant gas‐related events compared to air.

### 7.2 Mucosal Injuries

Inadvertent mucosotomy occurs in approximately 3.9%–10% of POEM cases. Minor mucosotomies can be closed with endoclips during POEM without compromising the procedure. Larger defects require more aggressive closure and may need endoscopic sutures or a tacking system. Unrecognized mucosotomy at the incision site risks mediastinitis or peritonitis; any suspected entry‐site dehiscence post‐procedure warrants prompt endoscopic reassessment.

### 7.3 Bleeding

Intraprocedural bleeding is inherent to TSE, given the rich submucosal vasculature, particularly on the gastric side, where larger perforator vessels are consistently encountered (and can be used as anatomical landmarks). Prevention relies on pre‐coagulation of visible vessels using the coagulation grasper (soft coagulation mode, 80 W) before transection, meticulous hemostasis throughout tunneling, and careful avoidance of vessels on the mucosal undersurface. Active bleeding is managed with irrigation, cap tamponade during instrument changes, or a coagulation grasper applied to both ends of the divided vessels. Temporary systolic blood pressure reduction below 100 mmHg, coordinated with anesthesia, facilitates hemostasis during brisk bleeding from hypercarbia‐related hypertension.

### 7.4 Post‐Procedural Infectious Complications

A single perioperative dose of intravenous antibiotics followed by at least 3 days of IV/oral antibiotics is recommended for all TSE procedures. Persistent fever, chest pain, or leukocytosis beyond 48 h should be evaluated with CT chest to exclude mediastinitis, tunnel hematoma, or pneumonia. Persistent abdominal pain beyond 2 days post‐G‐POEM warrants re‐assessment.

## 8 Conclusion

Third‐space endoscopy has fundamentally transformed the management of benign gastrointestinal motility disorders. From the index application of POEM for achalasia—now one of the most thoroughly studied procedures in advanced endoscopy with clinical success exceeding 90% at 7 years and endorsement from all major GI societies—to the expanding portfolio of Z‐POEM, D‐POEM, G‐POEM, PREM, and POEM+F, the SEMF principle has consistently demonstrated versatility, safety, and reproducibility across the GI tract. The technique of POEM has evolved considerably since its inception: shorter myotomies, selective circular muscle dissection, EndoFLIP‐guided endpoints, and anti‐reflux innovations such as SP‐POEM and POEM+F have led to better results.

Post‐POEM GER remains the principal unresolved challenge. For TSE indications beyond achalasia cardia, careful patient selection informed by comprehensive physiological assessment—HRM, EndoFLIP, timed barium esophagram—and performance at experienced centers will continue to refine the evidence base and optimize outcomes.

Looking ahead, artificial intelligence‐assisted HRM interpretation, robotic flexible endoscopy platforms, and integrated intraoperative physiologic feedback systems will define the next era of TSE—one in which the endoscopist, operating in the ‘third space’, increasingly assumes the role of a precision interventionist for conditions once exclusively in the surgical domain.

## Author Contributions

**Sanjana Bhagwat**: conceptualization, writing – original draft, methodology, validation, visualization, writing – review and editing, and investigation. **Amol Bapaye**: conceptualization, investigation, writing – review and editing, supervision, validation, methodology, visualization, and resources.

## Funding

The authors have nothing to report.

## Ethics Statement

**Approval of the research protocol by an Institutional Review Board**: N/A.

## Consent

N/A.

## Conflicts of Interest

The authors declare no conflicts of interest.
